# Generalization of the space $l(p)$ derived by absolute Euler summability and matrix operators

**DOI:** 10.1186/s13660-018-1724-9

**Published:** 2018-06-15

**Authors:** Fadime Gökçe, Mehmet Ali Sarıgöl

**Affiliations:** Department of Mathematics, University of Pamukkale, Denizli, Turkey

**Keywords:** 40C05, 40F05, 46A45, Absolute summability, Euler means, Matrix transformations, Sequences spaces

## Abstract

The sequence space $l(p)$ having an important role in summability theory was defined and studied by Maddox (Q. J. Math. 18:345–355, 1967). In the present paper, we generalize the space $l(p)$ to the space $\vert E_{\phi }^{r} \vert (p)$ derived by the absolute summability of Euler mean. Also, we show that it is a paranormed space and linearly isomorphic to $l(p)$. Further, we determine *α*-, *β*-, and *γ*-duals of this space and construct its Schauder basis. Also, we characterize certain matrix operators on the space.

## Introduction

Let *X*, *Y* be any subsets of *ω*, the set of all sequences of complex numbers, and $A=(a_{nv})$ be an infinite matrix of complex numbers. By $A(x)=(A_{n}(x))$, we indicate the *A*-transform of a sequence $x= ( x_{v} ) $ if the series

$$ A_{n} ( x ) =\sum_{v=0}^{\infty }a_{nv}x_{v} $$

are convergent for $n\geq 0$. If $Ax\in Y$, whenever $x\in X$, then *A*, denoted by $A:X\rightarrow Y$, is called a matrix transformation from *X* into *Y*, and we mean the class of all infinite matrices *A* such that $A:X\rightarrow Y$ by $(X,Y)$. For $c_{s}$, $b_{s}$, and $l_{p}$ ($p\geq 1$), we write the space of all convergent, bounded, *p*-absolutely convergent series, respectively. Further, the matrix domain of an infinite matrix *A* in a sequence space *X* is defined by

1 $$ X_{A}= \bigl\{ x= ( x_{n} ) \in \omega :A(x)\in X \bigr\} . $$

The *α*-, *β*-, and *γ*-duals of the space *X* are defined as follows:

$$\begin{aligned}& X^{\alpha }= \bigl\{ \epsilon \in \omega :(\epsilon_{n}x_{n}) \in l_{1} \text{ for all } x\in X \bigr\} , \\ & X^{\beta }= \bigl\{ \epsilon \in \omega :(\epsilon_{n}x_{n}) \in c_{s} \text{ for all } x\in X \bigr\} , \\ & X^{\gamma }= \bigl\{ \epsilon \in \omega :(\epsilon_{n}x_{n}) \in b_{s} \text{ for all } x\in X \bigr\} . \end{aligned}$$

A subspace *X* is called an *FK* space if it is a Frechet space, that is, a complete locally convex linear metric space, with continuous coordinates $P_{n}:X\rightarrow C$ ($n=1,2,\ldots $), where $P_{n}(x)=x_{n}$ for all $x\in X$; an *FK* space whose metric is given by a norm is said to be a *BK* space. An *FK* space *X* including the set of all finite sequences is said to have *AK* if

$$ \lim_{m\rightarrow \infty }x^{ [ m ] }=\lim_{m\rightarrow \infty }\sum _{v=0}^{m}x_{v}e^{(v)}=x $$

for every sequence $x\in X$, where $e^{(v)}$ is a sequence whose only non-zero term is one in *v*th place for $v\geq 0$. For example, it is well known that the Maddox space

$$ l(p)= \Biggl\{ x=(x_{n}):\sum_{n=1}^{\infty } \vert x_{n} \vert ^{p_{n}}< \infty \Biggr\} $$

is an *FK* space with *AK* with respect to its natural paranorm

$$ g(x)= \Biggl( \sum_{n=0}^{\infty } \vert x_{n} \vert ^{p_{n}} \Biggr) ^{1/M}, $$

where $M=\max \{ 1,\sup_{n}p_{n} \} $; also it is even a *BK* space if $p_{n}\geq 1$ for all *n* with respect to the norm

$$ \Vert x \Vert =\inf \Biggl\{ \delta >0:\sum_{n=0}^{\infty } \vert x_{n}/\delta \vert ^{p_{n}}\leq 1 \Biggr\} $$

([19–21, 29]).

Throughout this paper, we assume that $0<\inf p_{n}\leq H<\infty $ and $p_{n}^{\ast }$ is a conjugate of $p_{n}$, *i.e.*, $1/p_{n}+1/p_{n} ^{\ast }=1$, $p_{n}>1$, and $1/p_{n}^{\ast }=0$ for $p_{n}=1$.

Let $\sum a_{v}$ be a given infinite series with $s_{n}$ as its *n*th partial sum, $\phi = ( \phi_{n} ) $ be a sequence of positive real numbers and $p= ( p_{n} ) $ be a bounded sequence of positive real numbers. The series $\sum a_{v}$ is said to be summable $\vert A,\phi_{n} \vert ( p ) $ if (see [10])

$$ \sum_{n=1}^{\infty } ( \phi_{n} ) ^{p_{n}-1} \bigl\vert A _{n} ( s ) -A_{n-1} ( s ) \bigr\vert ^{p_{n}}< \infty . $$

It should be noted that the summability $\vert A,\phi_{n} \vert (p)$ includes some well-known summability methods for special cases of *A*, *ϕ* and $p=(p_{n})$. For example, if we take $A=E^{r}$ and $p_{n}=k$ for all *n*, then it is reduced to the summability method $\vert E,r \vert _{k}$ (see [12]) where Euler matrix $E^{r}$ is defined by

$$ e_{nk}^{r}= \textstyle\begin{cases} {{{n}}\choose {{k}}}(1-r)^{n-k}r^{k}, &0\leq k\leq n , \\ 0,& k>n , \end{cases} $$

for $0< r<1$ and

$$ e_{nk}^{1}= \textstyle\begin{cases} 0, &0\leq k< n , \\ 1, &k=n. \end{cases} $$

Also we refer the readers to the papers [7, 9, 30, 31, 35] for detailed terminology.

A large literature body, concerned with producing sequence spaces by means of matrix domain of a special limitation method and studying their algebraic, topological structure and matrix transformations, has recently grown. In this context, the sequence spaces $\overline{l}(p)$, $r_{p}^{t}$, $l(u,v,p)$, and $l(N^{t},p)$ were studied by Choudhary and Mishra [8], Altay and Başar [2, 3], Yeşilkayagil and Başar [37] by defining as the domains of the band, Riesz, the factorable, and Nörlund matrices in the $l(p)$ (see also [1, 4–6, 16–18, 23–28]).

Also, some series spaces have been derived and examined by various absolute summability methods from a different point of view (see [13, 14, 32, 34]). In this paper, we generalize the space $l(p)$ to the space $\vert E_{\phi }^{r} \vert (p)$ derived by the absolute summability of Euler means and show that it is a paranormed space linearly isomorphic to $l(p)$. Further, we determine *α*-, *β*-, and *γ*-duals of this space and construct its Schauder basis. Finally, we characterize certain matrix transformations on the space.

First, we remind some well-known lemmas which play important roles in our research.

## Needed lemmas

### Lemma 2.1

([11])

*Let* $p= ( p_{v} ) $ *and* $q= ( q_{v} ) $ *be any two bounded sequences of strictly positive numbers*.

(i) *If* $p_{v}>1$ *for all* *v*, *then* $A\in ( l(p),l _{1} ) $ *if and only if there exists an integer* $M>1$ *such that*
  2 $$ \sup \Biggl\{ \sum_{v=0}^{\infty } \biggl\vert \sum_{n \in K}a_{nv}M^{-1} \biggr\vert ^{p_{v}^{\ast }}:K\subset N \textit{ finite} \Biggr\} < \infty . $$
(ii) *If* $p_{v}\leq 1$ *and* $q_{v}\geq 1$ *for all* $v\in N$, *then* $A\in ( l(p),l(q) ) $ *if and only if there exists some* *M* *such that*
  $$ \sup_{v}\sum_{n=0}^{\infty } \bigl\vert a_{nv}M^{-1/p_{v}} \bigr\vert ^{q_{n}}< \infty . $$
(iii) *If* $p_{v}\leq 1$, *then* $A\in ( l(p),c ) $ *if and only if*
  $$ (\mathrm{a})\quad \lim_{n}a_{nv}\textit{ exists for each }v,\quad\quad (\mathrm{b})\quad \sup_{n,v} \vert a_{nv} \vert ^{p_{v}}< \infty , $$
  *and* $A\in ( l(p),l_{\infty } ) $ *iff* (b) *holds*.
(iv) *If* $p_{v}>1$ *for all* *v*, *then* $A\in ( l(p),c ) $ *if and only if* (a) (a)* holds*, *and* (b)* there is a number* $M>1 $ *such that*
  $$ \sup_{n}\sum_{v=0}^{\infty } \bigl\vert a_{nv}M^{-1} \bigr\vert ^{p_{v}^{\ast }}< \infty , $$
  *and* $A\in ( l(p),l_{\infty } ) $ *iff* (b) *holds*.

It may be noticed that condition (2) exposes a rather difficult condition in applications. The following lemma produces a condition to be equivalent to (2) and so the following lemma, which is more practical in many cases, will be used in the proofs of theorems.

### Lemma 2.2

([33])

*Let* $A= ( a_{nv} ) $ *be an infinite matrix with complex numbers and* $( p_{v} ) $ *be a bounded sequence of positive numbers*. *If* $U_{p} [ A ] < \infty $ *or* $L_{p} [ A ] <\infty $, *then*

$$ ( 2C ) ^{-2}U_{p} [ A ] \leq L_{p} [ A ] \leq U_{p} [ A ] , $$

*where* $C=\max \{ 1,2^{H-1} \} $, $H=\sup _{v}p_{v}$,

$$ U_{p} [ A ] =\sum_{v=0}^{\infty } \Biggl( \sum_{n=0}^{\infty } \vert a_{nv} \vert \Biggr) ^{p_{v}} $$

*and*

$$ L_{p} [ A ] =\sup \Biggl\{ \sum_{v=0}^{\infty } \biggl\vert \sum_{n\in K}a_{nv} \biggr\vert ^{p_{v}}:K\subset N\textit{ finite} \Biggr\} . $$

### Lemma 2.3

([22])

*Let* *X* *be an* *FK* *space with* *AK*, *T* *be a triangle*, *S* *be its inverse*, *and* *Y* *be an arbitrary subset of ω*. *Then we have* $A\in ( X_{T},Y ) $ *if and only if* $\widehat{A}\in ( X,Y ) $ *and* $V^{(n)}\in ( X,c ) $ *for all* *n*, *where*

$$ \hat{a}_{nv}=\sum_{j=v}^{\infty }a_{nj}s_{jv};\quad n,v=0,1, \ldots, $$

*and*

$$ v_{mv}^{(n)}= \textstyle\begin{cases} \sum_{j=v}^{m}a_{nj}s_{jv},&0\leq v\leq m, \\ 0, &v>m. \end{cases} $$

## Main theorems

In this section, we introduce the paranormed series space $\vert E _{\phi }^{r} \vert (p)$ as the set of all series summable by the absolute summability method of Euler matrix and show that this space is linearly isomorphic to the space $l(p)$. Also, we compute the Schauder base, *α*-, *β*-, and *γ*-duals of the space and characterize certain matrix transformations defined on that space.

First of all, we note that, by the definition of the summability $\vert A,\phi_{n} \vert (p)$, we can write the space $\vert E_{\phi }^{r} \vert (p)$ as

$$ \bigl\vert E_{\phi }^{r} \bigr\vert (p)= \Biggl\{ a\in \omega :\sum_{n=0}^{\infty }\phi_{n}^{p_{n}-1} \bigl\vert \bigtriangleup A _{n}^{r} ( s ) \bigr\vert ^{p_{n}}< \infty \Biggr\} , $$

where

$$ \bigtriangleup A_{n}^{r} ( s ) =A_{n}^{r}(s)-A_{n-1}^{r}(s) $$

and

$$ A_{n}^{r}(s)=\sum_{k=0}^{n} {{{n}}\choose {{k}}}(1-r)^{n-k}r^{k}s _{k},\quad n \geq 0, \quad\quad A_{-1}^{r}(s)=0. $$

Also, a few calculations give

$$\begin{aligned} \bigtriangleup A_{n}^{r} ( s ) = & \sum _{m=0}^{n} \sum_{k=m}^{n} {{{n}}\choose {{k}}}(1-r)^{n-k}r^{k}a_{m}-\sum _{m=0}^{n-1}\sum_{k=m}^{n-1} {{{n-1}}\choose {{k}}}(1-r)^{n-1-k}r ^{k}a_{m} \\ = & \sum_{m=1}^{n}\sum _{k=m}^{n}(1-r)^{n-1-k} \biggl[ {{{n-1}}\choose {{k-1}}}-r{{{n}}\choose {{k}}} \biggr] r^{k}a_{m} \\ = & \sum_{m=1}^{n}\sigma_{nm}a_{m}, \end{aligned}$$

where

$$ \sigma_{nm}= \textstyle\begin{cases} \sum_{k=m}^{n}(1-r)^{n-1-k}r^{k} [ {{{n-1}}\choose {{k-1}}}-r{{{n}}\choose {{k}}} ] , &1\leq m\leq n , \\ 0, & m>n. \end{cases} $$

Further, it follows by putting $r=q(1+q)^{-1}$

$$\begin{aligned} \sigma_{nm} = & (1+q)^{1-n}\sum _{k=m}^{n}q^{k} \biggl[ {{{n-1}}\choose {{k-1}}}-q(1+q)^{-1}{{{n}}\choose {{k}}} \biggr] \\ & \\ = & (1+q)^{-n}\sum_{k=m}^{n} \biggl[ q^{k} {{{n-1}}\choose {{k-1}}}-q^{k+1} {{{n-1}}\choose {{k}}} \biggr] \\ & \\ = & q^{m}(1+q)^{-n}{{{n-1}}\choose {{m-1}}}= {{{n-1}}\choose {{m-1}}}(1-r)^{n-m}r ^{m}. \end{aligned}$$

Now, by considering $T_{n}^{r}(\phi ,p)(a)=\phi_{n}^{1/{p_{n}^{\ast }}} \bigtriangleup A_{n}^{r} ( s ) $, we immediately get that $T_{0}^{r}(\phi ,p)(a)=a_{0}\phi_{0}^{1/{p_{0}^{\ast }}}$ and

3 $$\begin{aligned} T_{n}^{r}(\phi ,p) (a) =&\phi_{n}^{1/{p_{n}^{\ast }}} \sum_{k=1}^{n}{{{n-1}}\choose {{k-1}}}(1-r)^{n-k}r^{k}a_{k} \\ =&\sum_{k=1}^{n}t_{nk}^{r}( \phi ,p)a_{k}, \end{aligned}$$

where

4 $$ t_{nk}^{r}(\phi ,p)= \textstyle\begin{cases} \phi_{0}^{1/{p_{0}^{\ast }}}, &k=n=0 , \\ \phi_{n}^{1/{p_{n}^{\ast }}}{{{n-1}}\choose {{k-1}}}(1-r)^{n-k}r^{k}, & 1\leq k\leq n, \\ 0, &k>n. \end{cases} $$

Therefore, we can state the space $\vert E_{\phi }^{r} \vert (p)$ as follows:

$$ \bigl\vert E_{\phi }^{r} \bigr\vert (p)= \Biggl\{ a=(a_{k}):\sum_{n=1}^{\infty } \Biggl\vert \phi_{n}^{1/{p_{n}^{\ast }}}\sum_{k=1}^{n} {{{n-1}}\choose {{k-1}}}(1-r)^{n-k}r^{k}a_{k} \Biggr\vert ^{p_{n}}< \infty \Biggr\} , $$

or

$$ \bigl\vert E_{\phi }^{r} \bigr\vert (p)= \bigl[ l(p) \bigr] _{T^{r}( \phi ,p)} $$

according to notation (1).

Further, since every triangle matrix has a unique inverse which is a triangle (see [36]), the matrix $T^{r}(\phi ,p)$ has a unique inverse $S^{r}(\phi ,p)=(s_{nk}^{r}(\phi ,p))$ given by

5 $$\begin{aligned} s_{nk}^{r}(\phi ,p)= \textstyle\begin{cases} \phi_{0}^{-1/{p_{0}^{\ast }}}, &k=n=0 , \\ \phi_{k}^{-1/{p_{k}^{\ast }}}{{{n-1}}\choose {{k-1}}}(r-1)^{n-k}r^{-n}, &1\leq k\leq n , \\ 0, &k>n. \end{cases}\displaystyle \end{aligned}$$

Before main theorems, note that if $r=1$ and $\phi_{n}=1$ for all $n\geq 0$, the space $\vert E_{\phi }^{r} \vert (p)$ is reduced to the space $l(p)$.

### Theorem 3.1

*Let* $0< r<1$ *and* $p=(p_{n})$ *be a bounded sequence of non*-*negative numbers*. *Then*:

- *The set* $\vert E_{\phi }^{r} \vert (p)$ *becomes a linear space with the coordinate*-*wise addition and scalar multiplication*, *and also it is an* *FK*-*space with respect to the paranorm*
  $$ \Vert x \Vert _{ \vert E_{\phi }^{r} \vert (p)}= \Biggl( \sum_{n=0}^{\infty } \bigl\vert T_{n}^{r}(\phi ,p) (x) \bigr\vert ^{p_{n}} \Biggr) ^{1/M}, $$
  *where* $M=\max \{ 1,\sup p_{n} \} $.
- *The space* $\vert E_{\phi }^{r} \vert (p)$ *is linearly isomorphic to the space* $l(p)$, *i*.*e*., $\vert E_{\phi }^{r} \vert (p) \cong l(p)$.
- *Define a sequence* $(b_{n}^{(v)})$ *by* $S^{r} ( (e^{(v)}) ) = ( \sum_{v=0}^{n}s_{nv}^{r}(\phi ,p)e^{(v)} ) $. *Then the sequence* $(b_{n}^{(v)})$ *is the Schauder base of the space* $\vert E_{\phi }^{r} \vert (p)$.
- *The space* $\vert E_{\phi }^{r} \vert (p)$ *is separable*.

### Proof

(a) The first part is a routine verification, so it is omitted. Since $T^{r}(\phi ,p)$ is a triangle matrix and $l(p)$ is an *FK*-space, it follows from Theorem 4.3.2 in [36] that $\vert E_{\phi }^{r} \vert (p)= [ l(p) ] _{T^{r}( \phi ,p)}$ is an *FK*-space.

(b) We should show that there exists a linear bijection between the spaces $\vert E_{\phi }^{r} \vert (p)$ and $l(p)$. Now, consider $T^{r}(\phi ,p): \vert E_{\phi }^{r} \vert (p) \rightarrow l(p)$ given by (3). Since the matrix corresponding this transformation is a triangle, it is obvious that $T^{r}(\phi ,p)$ is a linear bijection. Furthermore, since $T^{r}(\phi ,p)(x)\in l(p)$ for $x\in \vert E_{\phi }^{r} \vert (p)$, we get

$$ \Vert x \Vert _{ \vert E_{\phi }^{r} \vert (p)}= \Biggl( \sum_{n=0}^{\infty } \bigl\vert T_{n}^{r}(\phi ,p) (x) \bigr\vert ^{p_{n}} \Biggr) ^{1/M}= \bigl\Vert T^{r}(\phi ,p) (x) \bigr\Vert _{l(p)}. $$

So, $T^{r}(\phi ,p)$ preserves the paranorm, which completes this part of the proof.

(c) Since the sequence $(e^{(v)})$ is the Schauder base of the space $l(p)$ and $\vert E_{\phi }^{r} \vert (p)= [ l(p) ] _{T^{r}(\phi ,p)}$, it can be written from Theorem 2.3 in [15] that $b^{(v)}=(S^{r}(\phi ,p)(e^{(v)}))$ is a Schauder base of the space $\vert E_{\phi }^{r} \vert (p)$.

(d) Since the space $\vert E_{\phi }^{r} \vert (p)$ is a linear metric space with a Schauder base, it is separable. □

### Theorem 3.2

*Let* $0< r<1$. *Define*

$$\begin{aligned}& D_{1}^{r}= \Biggl\{ a\in \omega :\exists M>1, \sum _{v=0}^{\infty } \Biggl( \sum_{n=v}^{\infty } \bigl\vert M^{-1}b_{n}^{(v)}a_{n} \bigr\vert \Biggr) ^{p_{v}^{\ast }}< \infty \Biggr\} , \\& D_{2}^{r}= \Biggl\{ a\in \omega :\exists M>1,\sup _{v}M^{1/p_{v}}\sum_{n=v}^{\infty } \bigl\vert b_{n}^{(v)}a_{n} \bigr\vert < \infty \Biggr\} , \\& D_{3}^{r}= \Biggl\{ a\in \omega :\sum _{n=v}^{\infty }b_{n}^{(v)}a_{n} \textit{ converges for each } v \Biggr\} , \\& D_{4}^{r}= \Biggl\{ a\in \omega : \exists M>1, \sup _{n}\sum_{v=1} ^{n} \Biggl\vert \sum_{k=v}^{n}b_{k}^{(v)}a_{k}M^{-1} \Biggr\vert ^{p_{v}^{\ast }}< \infty \Biggr\} , \\& D_{5}^{r}= \Biggl\{ a\in \omega :\sup_{n,v} \Biggl\vert \sum_{k=v} ^{n}b_{k}^{(v)}a_{k} \Biggr\vert ^{p_{v}}< \infty \Biggr\} . \end{aligned}$$

(i) *If* $p_{v}>1$ *for all* *v*, *then*
  $$ \bigl\{ \bigl\vert E_{\phi }^{r} \bigr\vert (p) \bigr\} ^{\alpha }=D _{1}^{r},\quad\quad \bigl\{ \bigl\vert E_{\phi }^{r} \bigr\vert (p) \bigr\} ^{ \beta }=D_{4}^{r} \cap D_{3}^{r}, \quad\quad \bigl\{ \bigl\vert E_{\phi }^{r} \bigr\vert (p) \bigr\} ^{\gamma }=D_{4}^{r}. $$
(ii) *If* $p_{v}\leq 1$ *for all* *v*, *then*
  $$ \bigl\{ \bigl\vert E_{\phi }^{r} \bigr\vert (p) \bigr\} ^{\alpha }=D _{2}^{r}, \quad\quad \bigl\{ \bigl\vert E_{\phi }^{r} \bigr\vert (p) \bigr\} ^{ \beta }=D_{5}^{r} \cap D_{3}^{r},\quad\quad \bigl\{ \bigl\vert E_{\phi }^{r} \bigr\vert (p) \bigr\} ^{\gamma }=D_{5}^{r}. $$

### Proof

To avoid the repetition of a similar statement, we only calculate *β*-duals of $\vert E_{\phi }^{r} \vert (p)$.

(i) Let us recall that $a\in \{ \vert E_{\phi }^{r} \vert (p) \} ^{\beta }$ if and only if $ax\in cs$ whenever $x\in \vert E_{\phi }^{r} \vert (p)$. Now, by using (5), it can be obtained that

$$\begin{aligned} \sum_{k=0}^{n}a_{k}x_{k} = & T_{0}^{r}(\phi ,p) (x)\phi_{0} ^{-1/p_{0}^{\ast }}a_{0}+\sum_{k=1}^{n}a_{k} \sum_{v=1} ^{k}\phi_{v}^{-1/p_{v}^{\ast }} {{{k-1}}\choose {{v-1}}} ( r-1 ) ^{k-v}r^{-k}T_{v}^{r}( \phi ,p) (x) \\ = & T_{0}^{r}(\phi ,p) (x)\phi_{0}^{-1/p_{0}^{\ast }}a_{0}+ \sum_{v=1}^{n}\phi_{v}^{-1/p_{v}^{\ast }}T_{v}^{r}( \phi ,p) (x) \sum_{k=v}^{n}a_{k} {{{k-1}}\choose {{v-1}}} ( r-1 ) ^{k-v}r^{-k} \\ = & \sum_{v=0}^{n}d_{nv}T_{v}^{r}( \phi ,p) (x), \end{aligned}$$

where $D=(d_{nv})$ is defined by

$$ d_{nv} = \textstyle\begin{cases} \phi_{0}^{-1/p_{0}^{\ast }}a_{0},&n=v=0 , \\ \sum_{k=v}^{n}b_{k}^{(v)}a_{k},&1\leq v\leq n , \\ 0, &v>n. \end{cases} $$

Since $T^{r}(\phi ,p)(x)\in l(p)$ whenever $x\in \vert E_{\phi } ^{r} \vert (p)$, $a\in \{ \vert E_{\phi }^{r} \vert (p) \} ^{\beta }$ if and only if $D\in (l(p),c)$. So, it follows from Lemma 2.1 that $a\in D_{4} ^{r}\cap D_{3}^{r}$ if $p_{v}>1$ for all *v*, and also $a\in D_{5} ^{r}\cap D_{3}^{r}$ if $p_{v}\leq 1$ for all *v*.

The remaining part of the theorem can be similarly proved by Lemma 2.1. □

### Theorem 3.3

*Let* $A= ( a_{nv} ) $ *be an infinite matrix of complex numbers*, $( \phi_{n} ) $ *and* $( \psi _{n} ) $ *be sequences of positive numbers*, $p= ( p_{n} ) $ *and* $q= ( q_{n} ) $ *be arbitrary bounded sequences of positive numbers with* $p_{n}\leq 1$ *and* $q_{n}\geq 1$ *for all n*. *Further*, *let the matrix* *Â* *be defined by*

$$ \hat{a}_{nv}=\sum_{j=v}^{\infty }a_{nj}b_{j}^{(v)} $$

*and* $F=T^{r}(\psi ,q)\hat{A}$. *Then* $A\in ( \vert E_{\phi } ^{r} \vert (p), \vert E_{\psi }^{r} \vert (q) ) $ *if and only if there exists an integer* $M>1$ *such that*, *for* $n=0,1,\ldots$ ,

6 $$\begin{aligned}& \sum_{k=v}^{\infty }b_{k}^{(v)}a_{nk} \quad \textit{converges for each } v, \end{aligned}$$

7 $$\begin{aligned}& \sup_{m,v} \Biggl\vert \sum_{k=v}^{m}b_{k}^{(v)}a_{nk} \Biggr\vert ^{p_{v}}< \infty , \end{aligned}$$

*and*

8 $$\begin{aligned} \sup_{v}\sum_{n=0}^{\infty } \bigl\vert M^{-1/p_{v}}f_{nv} \bigr\vert ^{q_{n}}< \infty . \end{aligned}$$

### Proof

Suppose that $p_{v}\leq 1$, $q_{v}\geq 1$ for all *v*. Note that $\vert E_{\phi }^{r} \vert (p)= [ l(p) ] _{T^{r}(\phi ,p)}$ and $\vert E_{\psi }^{r} \vert (q)= [ l(q) ] _{T^{r}(\psi ,q)}$. By Lemma 2.3, $A\in ( \vert E _{\phi }^{r} \vert (p), \vert E_{\psi }^{r} \vert (q) ) $ if and only if $\hat{A}\in ( l(p), \vert E_{\psi }^{r} \vert (q) ) $ and $V^{(n)} \in ( l(p),c ) $, where the matrix $V^{(n)}$ is defined by

$$ v_{mv}^{(n)}= \textstyle\begin{cases} \sum_{j=v}^{m}b_{j}^{(v)}a_{nj},&0\leq v\leq m, \\ 0,& v>m. \end{cases} $$

One can see that since $\hat{A}(x)\in \vert E_{\psi }^{r} \vert (q)= [ l(q) ] _{T^{r}(\psi ,q)}$ whenever $x\in l(p)$, $\hat{A}\in ( l(p), \vert E_{\psi }^{r} \vert (q) ) $ iff $F=T^{r}(\psi ,q)\hat{A}\in ( l(p),l(q) ) $. Now, applying Lemma 2.1(ii) and (iii) to the matrices *F* and $V^{(n)}$, it follows that $V^{(n)}\in ( l(p),c ) $ iff, for $n=0,1,\ldots$ , conditions (6) and (7) hold, and $F\in ( l(p),l(q) ) $ iff there exists an integer *M* such that

$$ \sup_{v}\sum_{n=0}^{\infty } \bigl\vert M^{-1/p_{v}}f _{nv} \bigr\vert ^{q_{n}}< \infty , $$

which completes the proof. □

### Theorem 3.4

*Assume that* $A= ( a_{nv} ) $ *is an infinite matrix of complex numbers and* $( \phi_{n} ) $, $( \psi_{n} ) $ *are sequences of positive numbers*. *If* $p= ( p _{n} ) $ *is an arbitrary bounded sequence of positive numbers such that* $p_{n}>1$ *for all* *n*, *and* $H=T^{r}(\psi ,1)\hat{A}$, *then* $A\in ( \vert E_{\phi }^{r} \vert (p), \vert E_{ \psi }^{r} \vert (1) ) $ *if and only if there exists an integer* $M>1$ *such that*, *for* $n=0,1,\ldots$ ,

9 $$\begin{aligned}& \sum_{k=v}^{\infty }b_{k}^{(v)} a_{nk} \quad \textit{converges for each } v \end{aligned}$$

10 $$\begin{aligned}& \sup_{n}\sum_{v=0}^{\infty } \Biggl\vert \sum_{k=v}^{n}b _{k}^{(v)} a_{nk}M^{-1} \Biggr\vert ^{p_{v}^{\ast }}< \infty \end{aligned}$$

*and*

11 $$\begin{aligned} \sum_{v=0}^{\infty } \Biggl( \sum _{n=0}^{\infty } \bigl\vert M ^{-1}h_{nv} \bigr\vert \Biggr) ^{p_{v}^{\ast }}< \infty . \end{aligned}$$

### Proof

Let $p_{n}>1$ for all *n*. It is clear that $\vert E _{\phi }^{r} \vert (p)= [ l(p) ] _{T^{r}(\phi ,p)}$ and $\vert E_{\psi }^{r} \vert (1)=l_{T^{r}(\psi ,1)}$. So, by Lemma 2.3, we have $A\in ( \vert E_{\phi }^{r} \vert (p), \vert E_{\psi }^{r} \vert (1) ) $ if and only if $\hat{A}\in ( l(p), \vert E_{\psi }^{r} \vert (1) ) $ and $V^{(n)}\in ( l(p),c ) $, where *Â* and $V^{(n)}$ are given in Theorem 3.3. If we take $H=T^{r}(\psi ,1) \hat{A}$, then it is easily seen that $\hat{A}\in ( l(p), \vert E _{\psi }^{r} \vert (1) ) $ iff $H\in ( l(p),l_{1} ) $ because, if $\hat{A}(x)\in \vert E_{\psi }^{r} \vert (1)$ for all $x\in l_{1} ( p ) $, $H(x)=T^{r}(\psi ,1)(\hat{A}(x)) \in l_{1}$. So, applying Lemma 2.1(iv) to the matrix $V^{(n)}$, it is obtained that $V^{(n)}\in ( l ( p ) ,c ) $ iff conditions (9) and (10) are satisfied. Again, if we apply Lemma 2.1(i) and Lemma 2.2 to the matrix *H*, then we have $H\in ( l(p),l _{1} ) $ iff the last condition holds. □

## Conclusion

The sequence spaces defined as domains of Riesz, factorable, Nörlund and *S*-matrices in the spaces $l(p)$ and the space of series summable by the absolute Euler have been recently studied by several authors. In this paper, we have defined the new absolute Euler space $\vert E _{\phi }^{r} \vert (p)$ and investigated some topological and algebraic properties such as isomorphism, duals, base, and also characterized certain matrix transformations on that space. So, we have extended some well-known results.
